# *In silico* analyses of the involvement of GPR55, CB1R and TRPV1: response to THC, contribution to temporal lobe epilepsy, structural modeling and updated evolution

**DOI:** 10.3389/fninf.2024.1294939

**Published:** 2024-02-07

**Authors:** Amy L. Cherry, Michael J. Wheeler, Karolina Mathisova, Mathieu Di Miceli

**Affiliations:** ^1^Worcester Biomedical Research Group, School of Science and the Environment, University of Worcester, Worcester, United Kingdom; ^2^Sustainable Environments Research Group, School of Science and the Environment University of Worcester, Worcester, United Kingdom; ^3^School of Science and the Environment University of Worcester, Worcester, United Kingdom

**Keywords:** CB1R, TRPV1, endocannabinoid system, evolution, protein structure, phylogenetic tree, transcriptomics, bioinformatics

## Abstract

**Introduction:**

The endocannabinoid (eCB) system is named after the discovery that endogenous cannabinoids bind to the same receptors as the phytochemical compounds found in Cannabis. While endogenous cannabinoids include anandamide (AEA) and 2-arachidonoylglycerol (2-AG), exogenous phytocannabinoids include Δ-9 tetrahydrocannabinol (THC) and cannabidiol (CBD). These compounds finely tune neurotransmission following synapse activation, via retrograde signaling that activates cannabinoid receptor 1 (CB1R) and/or transient receptor potential cation channel subfamily V member 1 (TRPV1). Recently, the eCB system has been linked to several neurological diseases, such as neuro-ocular abnormalities, pain insensitivity, migraine, epilepsy, addiction and neurodevelopmental disorders. In the current study, we aim to: (i) highlight a potential link between the eCB system and neurological disorders, (ii) assess if THC exposure alters the expression of eCB-related genes, and (iii) identify evolutionary-conserved residues in CB1R or TRPV1 in light of their function.

**Methods:**

To address this, we used several bioinformatic approaches, such as transcriptomic (Gene Expression Omnibus), protein–protein (STRING), phylogenic (BLASTP, MEGA) and structural (Phyre2, AutoDock, Vina, PyMol) analyzes.

**Results:**

Using RNA sequencing datasets, we did not observe any dysregulation of eCB-related transcripts in major depressive disorders, bipolar disorder or schizophrenia in the anterior cingulate cortex, nucleus accumbens or dorsolateral striatum. Following *in vivo* THC exposure in adolescent mice, *GPR55* was significantly upregulated in neurons from the ventral tegmental area, while other transcripts involved in the eCB system were not affected by THC exposure. Our results also suggest that THC likely induces neuroinflammation following *in vitro* application on mice microglia. Significant downregulation of *TPRV1* occurred in the hippocampi of mice in which a model of temporal lobe epilepsy was induced, confirming previous observations. In addition, several transcriptomic dysregulations were observed in neurons of both epileptic mice and humans, which included transcripts involved in neuronal death. When scanning known interactions for transcripts involved in the eCB system (n = 12), we observed branching between the eCB system and neurophysiology, including proteins involved in the dopaminergic system. Our protein phylogenic analyzes revealed that CB1R forms a clade with CB2R, which is distinct from related paralogues such as sphingosine-1-phosphate, receptors, lysophosphatidic acid receptors and melanocortin receptors. As expected, several conserved residues were identified, which are crucial for CB1R receptor function. The anandamide-binding pocket seems to have appeared later in evolution. Similar results were observed for TRPV1, with conserved residues involved in receptor activation.

**Conclusion:**

The current study found that GPR55 is upregulated in neurons following THC exposure, while TRPV1 is downregulated in temporal lobe epilepsy. Caution is advised when interpreting the present results, as we have employed secondary analyzes. Common ancestors for CB1R and TRPV1 diverged from jawless vertebrates during the late Ordovician, 450 million years ago. Conserved residues are identified, which mediate crucial receptor functions.

## 1 Introduction

The endocannabinoid (eCB) system acts as a regulator during neurotransmission. Indeed, following synaptic activation, eCBs will be released in the post-synapse, which, in turn, will decrease neuronal activation (Lu and Mackie, 2016). Historically, anandamide (AEA) and 2-arachidonoylglycerol (2-AG) were discovered first and named after the receptors they involve, mimicking the effects observed after “cannabis” exposure (Lu and Mackie, 2016). Expression of the cannabinoid receptor type 1 (CB1R) is mostly confined to the central nervous system (Matsuda et al., 1990), while CB2R expression is considered to be mainly located at the periphery (Munro et al., 1993), although potential clinical applications of targeting CB2R in neurological disorders have been highlighted recently (Kibret et al., 2022), likely due to microglial expression of CB2R (Tanaka et al., 2020). Other known eCB receptors include transient receptor potential vanilloid 1 (TRPV1) and G protein-coupled receptor 55 (GPR55) (Kano et al., 2009). Furthermore, GPR18 (McHugh et al., 2010), GPR119 (Overton et al., 2006) and some PPARs [peroxisome proliferator-activated receptors, reviewed in 2016 (O’Sullivan, 2016)] have also been identified as potential eCB receptors.

Derived from the omega-6 arachidonic acid (C20:4 n-6), both AEA and 2-AG are endogenous ligands of CB1R and induce decreased neuronal excitation (Ameri et al., 1999; Wilson and Nicoll, 2001; Straiker et al., 2023). Both molecules seem to involve a retrograde mechanism, whereby synthesis of AEA or 2-AG is located in the post-synapse, followed by diffusion in the synaptic cleft to then act on pre-synaptic vesicular fusion (Wilson and Nicoll, 2001). However, non-retrograde signaling has also been observed, as well as neuron-astrocyte communication (Castillo et al., 2012). The biosynthesis of AEA involves activation of N-acyltransferase (NAT) and N-acylphosphatidylethanolamine-hydrolyzing phospholipase D (NAPE-PLD), while 2-AG requires both phospholipase C (PLC) and diacylglycerol lipase (DAGL) (Kano et al., 2009). Degradation of AEA is performed by activation of fatty acid amide hydrolase (FAAH) and 2-AG is broken down by monoacyl-glycerol lipase (MAGL) (Kano et al., 2009). Interestingly, other degradation pathways can be triggered through two other enzymes: cyclooxygenase and lipoxygenase (Kano et al., 2009). The biological activity and regional distributions of these enzymes, at both the organ- and cell-specific levels, have been summarized previously (Basavarajappa, 2007).

Recent evidence has pinpointed that dysfunctions of the eCB system may cause neuro-ocular abnormalities (Bainbridge et al., 2022), pain insensitivity (Habib et al., 2019), migraine (Juhasz et al., 2009), epilepsy (Ludányi et al., 2008), addiction (Herman et al., 2006; Agrawal et al., 2009; Proudnikov et al., 2010), metabolic disease (de Miguel-Yanes et al., 2011), obesity (Russo et al., 2007) and neurodevelopmental disorders (Lu et al., 2008; Jung et al., 2012; Siniscalco et al., 2013; Gomis-González et al., 2016; Servadio et al., 2016; Wei et al., 2016; Barchel et al., 2018; Karhson et al., 2018; Melancia et al., 2018; Aran et al., 2019; Zamberletti et al., 2019; Wu et al., 2020). Many clinical observations have been paralleled and explained by animal studies, often linking AEA and neurodevelopmental disorders. In a recent meta-analysis, medical cannabis, largely used in the form of either tetrahydrocannabinol (THC) or cannabidiol (CBD), was found to be efficient for the treatment of several neurological diseases, such as chronic pain, spasticity, substance use disorder, epilepsy, Tourette’s syndrome and Parkinson’s Disease (Bilbao and Spanagel, 2022). Thus, activation of the eCB by natural or synthetic agonists provides efficient clinical benefits, which could be explained by the negative feedback role of the eCB system in neurotransmission.

The most probable evolutionary origin of the eCB system was explained in 2006 (McPartland et al., 2006). In fact, different evolutionary origins have been discovered, depending on which eCB is scrutinized. TRPV1 and GPR55 arose rather recently, likely in mammals (McPartland et al., 2006). The oldest member of the eCB family, FAAH, was present in early eukaryotic cells, while NAPE-PLD arose in opisthokonts, which comprise both animals and fungi. Recent evidence has corroborated previous observations, as two FAAH isoforms have been studied in the legume *Medicago truncatula* (Arias-Gaguancela et al., 2023), in addition to the known expression in the angiosperm *Arabidopsis thaliana* (Haq and Kilaru, 2020). Furthermore, a duplication event likely induced the appearance of CB1R and CB2R in vertebrates from an invertebrate deuterostome ancestor (Elphick et al., 2003), in which the pre-CBR protein was responsible for axonal regulation (Egertová and Elphick, 2007).

In this article, we aim to:

(i) Assess the potential transcriptomic alterations of 13 specific eCB-related transcripts in epilepsy, major depressive disorder, bipolar disorder or schizophrenia.
(ii) Assess the impact of THC on these 13 specific eCB-related transcripts.
(iii) Identify paralogues of CB1R or TRPV1 to infer evolutionary ancestry.
(iv) Identify conserved residues in CB1R or TRPV1 that mediate receptor function.

## 2 Methods

### 2.1 *In silico* analysis of publicly available RNA sequencing datasets

Previously-published RNA sequencing datasets were retrieved from the Gene Expression Omnibus (GEO) repository (Edgar et al., 2002; Clough and Barrett, 2016). Briefly, GEO datasets were scanned for adequate output using specific search terms. Table 1 describes the datasets used in the current study, with corresponding accession links given in Supplementary Table S1. We focused on 13 specific genes from the eCB system (Supplementary Table S2). Supplementary material M1 summarizes all DESeq2 results of the present study.

**Table 1:** Detailed description of all RNA sequencing datasets retrieved for the present study.

Dataset	Specie	Publication	Data type	Norm needed?	Setting	Tissue	Regions	Intervention group	Control group	Behavioral testing performed prior to RNA-seq
GSE80655	Hs	Ramaker et al. (2017)	Raw aligned counts	Yes	*Post mortem*	Brain	AntC, NAC, DLS	MDD, BD or SCZ	Healthy	n/a
GSE189821	Mm	Zuo et al. (2022)	Raw aligned counts	Yes	*In vivo*	Brain	PFC, NAC, AntC, DLS, VTA	Intraperitoneal THC, 10 mg/kg/day for 21 days, P30-44 start, washout 14 days	Vehicle ethanol:tween:saline, 1:1:18	Yes
GSE116813	Mm	Jouroukhin et al. (2019)	Raw aligned counts	yes	*In vivo*	brain	HC	Subcutaneous THC, 8 mg/kg/day for 21 days, P30 start, washout 21 days	Vehicle ethanol:cremaphor:saline, 1:1:18	Yes
GSE70689	Mm	Juknat et al. (2013)	Raw aligned counts	No	*In vitro*	Microglia	-	THC, 10 μM, 2 h.	None, as final [ethanol] **≤** 0.1%	n/a
GSE77578	Mm	Srivastava et al. (2018)	Raw aligned counts	no	*In vivo*	Brain	HC	EP induced by single intraperitoneal pilocarpine injection	Non-epileptic (pilocarpine-naïve)	No
GSE190451	Hs	Chen et al. (2023)	Raw aligned counts	Yes	*Post mortem*	Brain	NeoC	EP (temporal lobe)	Non-epileptic patients (undergoing benign brain tumor surgery, healthy tissue collected)	n/a
GSE74150	Rn	Damasceno et al. (2020)	Raw aligned counts	Yes	*In vivo*	Brain	Inferior colliculus	EP (audiogenic)	Non-epileptic	No

GSE, GEO (gene expression omnibus) series experient; Hs, *Homo sapiens*; Mm, *Mus musculus*; Rn, *Rattus norvegicus*; NAC, nucleus accumbens, AntC, anterior cingulate; DLS, dorsolateral striatum; HC, hippocampus; NeoC, neocortex; PFC, prefrontal cortex; VTA, ventral tegmental area; MDD, major depressive disorder; BD, bipolar disorder; SCZ, schizophrenia; EP, epilepsy; THC, tetrahydrocannabinol; Norm, normalization; n/a, non-applicable. Please see Supplementary Table S1 for accession links.

Differential gene expression analysis was performed in the freeware R Studio version 4.0.3 (R Core Team, 2013; RStudio Team, 2020). Following the workflow described by Sanchis et al. (2021), we first retrieved count data, using the *GEOquery* package. If necessary, count data were then normalized using the *DESeq2* package. Differential expression analyzes were performed using the *DESeq2* package. Gene annotations were performed with either the *org.Hs.eg.bd* (*Homo sapiens*), *org.Mm.eg.db* (*Mus musculus*) or *AnnotationDbi* packages, as appropriate. Volcano plots were drawn with the *EnhancedVolcano* package. Gene ontology (over-representation analysis, ORA) was performed with the *GOplot* package using Biological Processes. Gene set enrichment analysis (GSEA) was performed with *clusterProfiler* and *enrichplot* packages. Scatterplot graphs were drawn using the *ggplot2* package. All of the above-mentioned packages are available under open access at the Comprehensive R Archive Network (CRAN) repository.^1^

### 2.2 Protein phylogenetics

Phylogenetic analyzes were performed in Mega X (Kumar et al., 2018) following alignment of FASTA sequences. Evolutional analysis was performed using the maximum likelihood method. Initial trees were obtained by applying Neighbor-Join (NJ) and BioNJ algorithms (Gascuel, 1997) to a matrix of pairwise distances estimated using the Jones-Taylor-Thornton (JTT)-based model (Jones et al., 1992). The final drawn trees are those with the highest log likelihoods. One hundred bootstraps (*r* = 100) were used to infer confidence in the output trees. BLASTP was used to scan different species with *Homo sapiens* as bait (reference sequence), available at the National Center for Biotechnology Information (NCBI).^2^

To search for conserved domains/residues, protein sequence (*Homo sapiens*) alignments was performed in Clustal Omega, available at the European Molecular Biology Laboratory – European Bioinformatics Institute (EMBL-EBI).^3^

### 2.3 Protein structural analyzes

Structural prediction of transmembrane protein topology (TMHMM, Transmembrane prediction using Hidden Markov Models) was performed from protein FASTA sequences using the platform provided by the Department of Health Technology at the Technical University of Denmark,^4^ which was made publicly available in 2001 (Krogh et al., 2001). Homology models were created using Phyre2 (Kelley and Sternberg, 2009; Kelley et al., 2015). For docking of anandamide into TRPV1, the Cryo-EM structure of human TRPV1 in complex with the analgesic drug SB-366791 (PDB: 8GFA) was used. The A chain of the multimer was used with ligand and phospholipids removed from the .pdb file. The anandamide ligand (CHEMBL15848) was retrieved from the ChEMBL webserver as a .csv file and converted to a .pdb file using the Online SMILES Translator and Structure File Generator and then to a .pdbqt file with merged non-polar hydrogens with AutoDockTools version 1.5.7. The ligand was docked into the receptor using AutoDock Vina 1.1.2 (Trott and Olson, 2010) with a docking grid 20 Å in length in the x, y, and z directions centered on the “tunnel” anandamide-binding pocket previously identified (Morales et al., 2022), flexible N438, Y555, Y554, Y487 and D708 side-chains and an exhaustiveness of 20. Structural analysis was performed by superposition in PyMol (Schrödinger and DeLano, 2020). A professional academic license was acquired for PyMol.

### 2.4 Protein–protein interaction networks

Protein–protein interaction networks (Szklarczyk et al., 2019) were predicted using the STRING (Search Tool for the Retrieval of Interacting Genes) database server,^5^ which contains both known and predicted protein interactions. Queries were pasted with either a single protein name or multiple names (as appropriate). When relevant, outer networks were searched for by expanding the nodes on display, using the built-in database tool, to reflect the immediate, intermediate and extended protein interactomes. For protein clustering within the networks, k mean clustering (based on centroids) was applied (Dalmaijer et al., 2022) directly in the server.

## 3 Results

### 3.1 Neurodevelopmental disorders and the eCB system: no change for 13 selected transcripts

**Figure 1:**
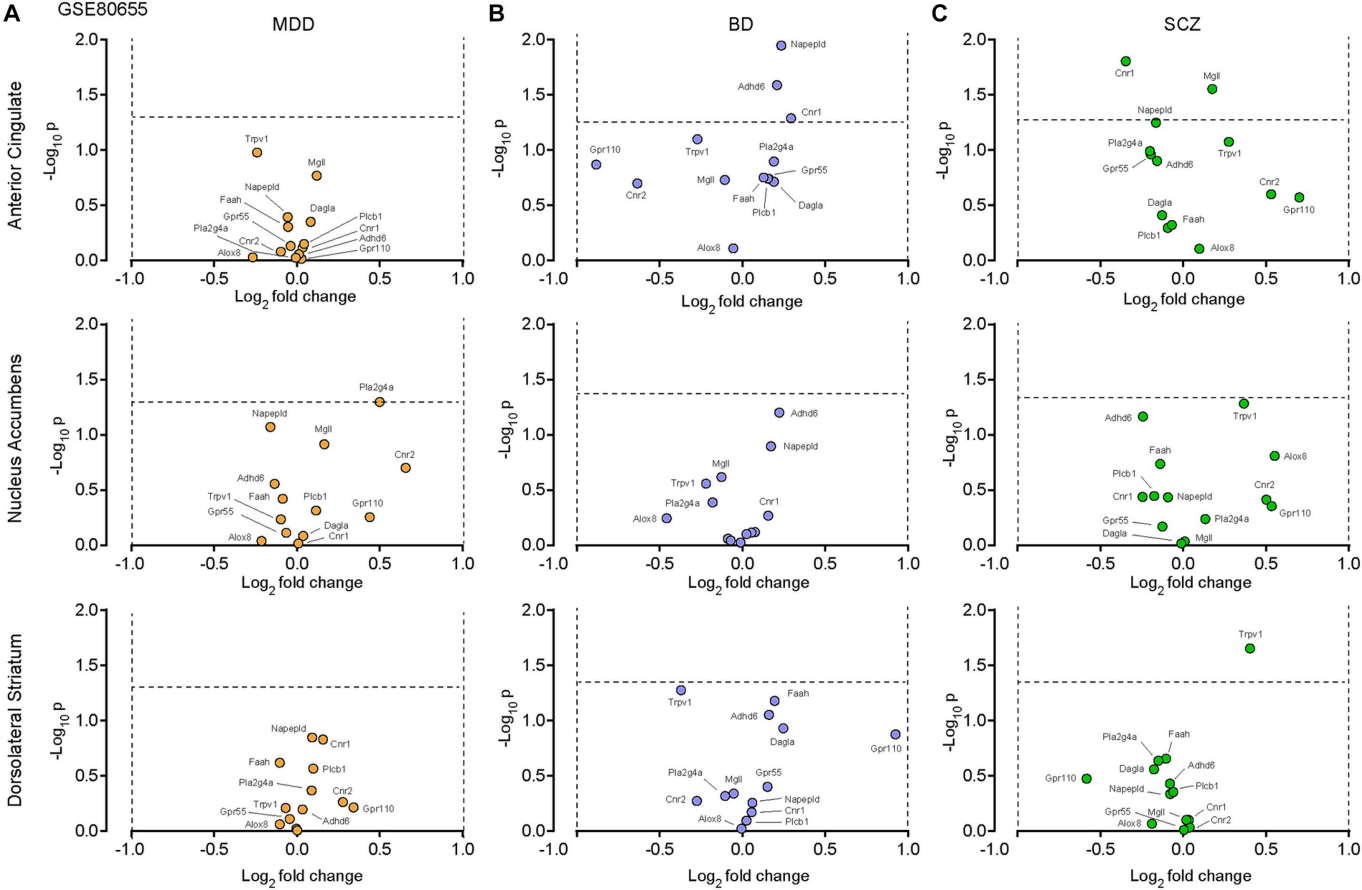
The eCB system in *postmortem* tissues from patients with major depressive disorders (MDD), bipolar disorder (BD) or schizophrenia (SCZ). Volcano plots of the RNA sequencing dataset GSE80655 (Ramaker et al., 2017), performed on neurons in the anterior cingulate cortex, nucleus accumbens and dorsolateral striatum comparing control patients to patients with clinically-determined MDD **(A)**, BD **(B)** or SCZ **(C)**. Twelve transcripts of interest, involved in the eCB system, were extracted from the GSE80655 dataset. The horizontal and vertical bars represent the significant threshold, set at *p* = 0.05 (−log_10_(p) = 1.3) and log_2_(fold change) >1 or < −1, respectively.

We first examined dataset GSE80655 (Ramaker et al., 2017), which contains RNA sequencing of 281 *postmortem* samples extracted from patients with either major depressive disorder (MDD, *n* = 69), bipolar disorder (BP, *n* = 71), schizophrenia (SCZ, *n* = 71) or controls (*n* = 70), in an attempt to decipher the potential role of the eCB system in these pathologies (Table 1). Out of 13 transcripts of interest (*CNR1*, *CNR2*, *GPR55*, *GPR110*, *TRPV1*, *MGLL*, *DAGLA*, *PLCB1*, *NAPEPLD*, *FAAH*, *ABHD6*, *PLA2G4A and ALOX8*, Supplementary Table S2), none were considered significantly up- or down-regulated in MDD (Figure 1A), respectively defined as log_2_(fold change) >1 or < −1 and -log_10_(p) ≥ 1.3 (*p* = 0.05). Similar results were observed with BP (Figure 1B) and SCZ (Figure 1C). These results indicate that our 13 transcripts of interest are not dysregulated under these conditions.

### 3.2 Chronic THC induces significant up-regulation of GPR55 in the ventral tegmental area of mice

**Figure 2:**
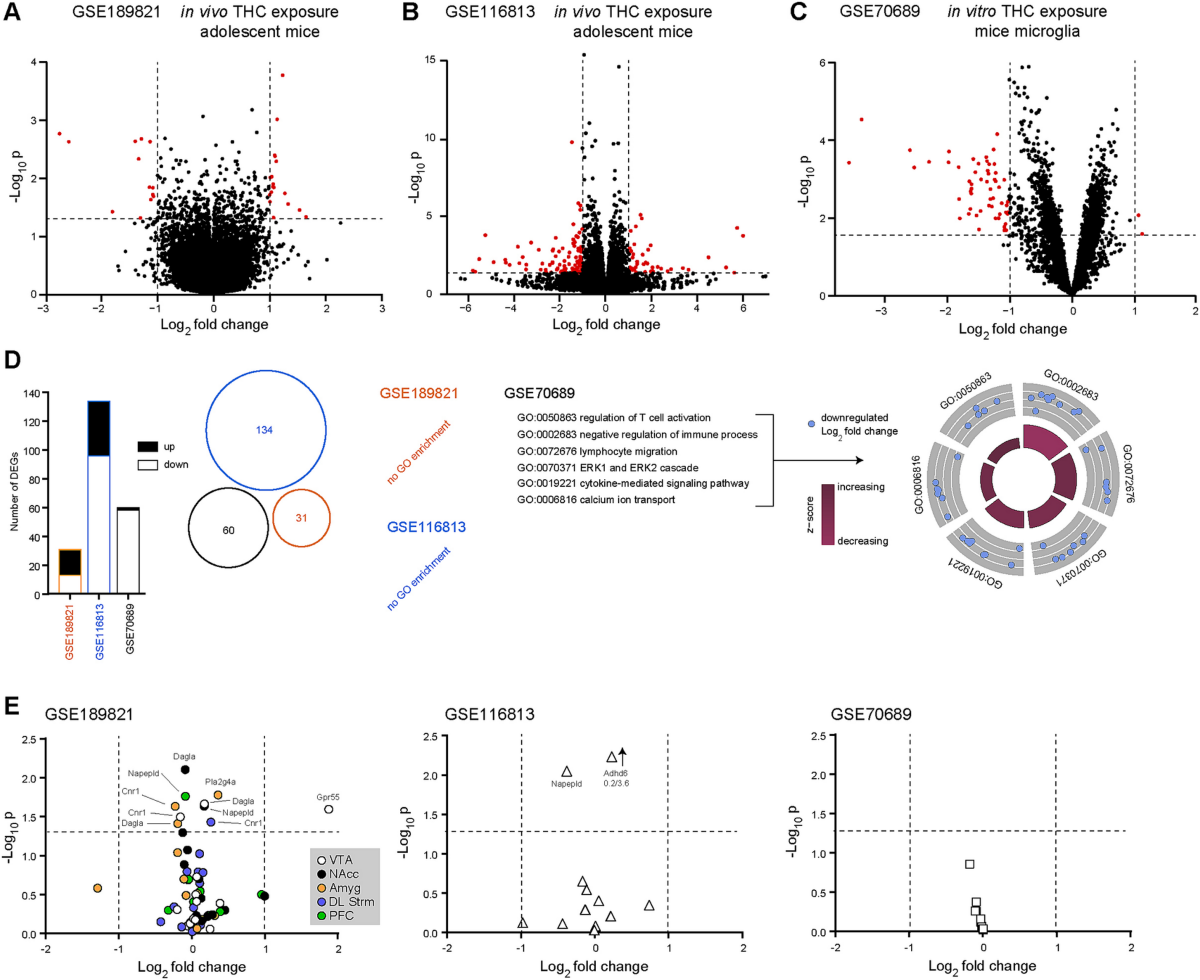
*GPR55* is significantly upregulated in the ventral tegmental area of adolescent mice following chronic *in vivo* THC exposure. **(A)** Volcano plot comparing transcript expression in adolescent mice chronically treated (3 weeks) with vehicle to THC (10 mg/kg/day), extracted from the GSE189821 dataset (Zuo et al., 2022). **(B)** Volcano plot comparing transcript expression in adolescent mice chronically treated (3 weeks) with vehicle to THC (8 mg/kg/day), extracted from the GSE116813 dataset (Jouroukhin et al., 2019). **(C)** Volcano plot comparing transcript expression in mice microglia treated *in vitro* with THC (10 μM for 2 h) or control (untreated), extracted from the GSE70689 dataset (Juknat et al., 2013). Red dots indicate significantly up- or down-regulated transcripts. Horizontal bars represent the significant threshold, set at *p* = 0.05 (−log_10_(p) = 1.3). vertical bars represent the threshold for significant differences in transcript expression, with either down- (<−1) or up- (>1) regulated transcripts. **(D)** Histograms (left), Venn diagram (middle) and gene ontology (biological processes) plots (right) showing no overlap between the significantly differentially expressed transcripts for the three datasets. In both GSE186821 and GSE116813, no biological processes were returned. However, in GSE70689, gene ontology (GO) analysis revealed several biological processes that are affected by THC exposure. Of interest, transcripts involved in neuro-inflammation and neurophysiology. **(E)** Out of the 12 transcripts of interest involved in the eCB system, only *GPR55* was significantly upregulated in the ventral tegmental area of adolescent mice exposed chronically to THC. Amyg, amygdala; DL Strm, dorsolateral striatum; NAcc, nucleus accumbens; PFC, prefrontal cortex; VTA, ventral tegmental area; GO, gene ontology; THC, Δ-9 tetrahydrocannabinol.

Next, we investigated whether we could detect transcriptional changes in our 13 transcripts of interest following acute or chronic THC exposure. To that end, we used GSE189821, GSE116813 and GSE70689 (Table 1). In GSE189821, 119 samples were analyzed from adolescent mice chronically exposed to THC (3 weeks at 10 mg/kg/day) or not (vehicle) and following a 14-day washout period, mimicking human behavior. In accordance with previous observations (Zuo et al., 2022), we found only minor transcriptomic dysregulations of our 13 eCB-related transcripts of interest following THC exposure in these mice (males and females pooled). This might be due to the long washout period that was applied. However, we noted 31 differentially expressed genes, with 13 transcripts down-regulated and 18 transcripts up-regulated, outside of our list of interest, when pooling all different brain regions together (Figure 2A). In the ventral tegmental area, *GPR55* was found to be significantly upregulated after THC exposure, the only one from our list of interest and out of all brain regions examined. In GSE116813, 12 hippocampal samples were sequenced from mice exposed to chronic THC (21 days at 8 mg/kg/day) or not (Jouroukhin et al., 2019) and after 21 days of washout. After subsetting the dataset to only include wild-type mice, we observed 134 differentially expressed transcripts, 38 up-regulated and 96 down-regulated (Figure 2B). As observed with the previous dataset, none of these differentially-expressed transcripts were linked to our list of interest. The next dataset, GSE70689, was used to observe acute transcriptional changes in mice microglia exposed to *in vitro* THC (10 μM for 2 h) (Juknat et al., 2013). Here, we performed subsetting of microglia exposed to either THC or vehicle, excluding samples exposed to cannabidiol. Using such a method, 60 transcripts were found to be significantly differentially expressed, 2 up-regulated and 58 downregulated (Figure 2C). None of our transcripts of interest were found to be significantly dysregulated in such a dataset. A further analysis revealed that none of the previously differentially expressed genes (outside our 13 transcripts of interest) overlapped in the three datasets (Venn diagram in Figure 2D). In addition to these observations, we also ran ORA Gene Ontology (GO) analyzes for each dataset. GSE189821 and GSE116813 returned no GO enrichment, while GSE70689 revealed many GO enrichments. These included transcripts involved in immune response, such as T cell activation, lymphocyte migration, cytokine-mediated signaling and ERK (extracellular signal-regulated kinases) cascades (Figure 2D). These surprising results indicate that our 13 transcripts of interest are only minimally involved in the response to *in vivo* THC exposure, at least in mice. However, *in vitro* exposure to THC likely activates microglia, in an attempt to prevent THC-induced inflammation. Representations of the three datasets (GSE189821, GSE116813 and GSE70689) and our 13 transcripts of interest can be found on Figure 2E. Note that *GPR55* is the only transcript of interest that was significantly upregulated after chronic *in vivo* THC exposure in mice, and only in the VTA (Figure 2E). This suggests that our 13 transcripts of interest were not affected by THC exposure, at least under these specific conditions, except for GPR55. GSEA analyzes did not reveal any biological processes of interest (not shown).

### 3.3 *TPRV1* is significantly downregulated in the epileptic tissue of patients with temporal lobe epilepsy

**Figure 3:**
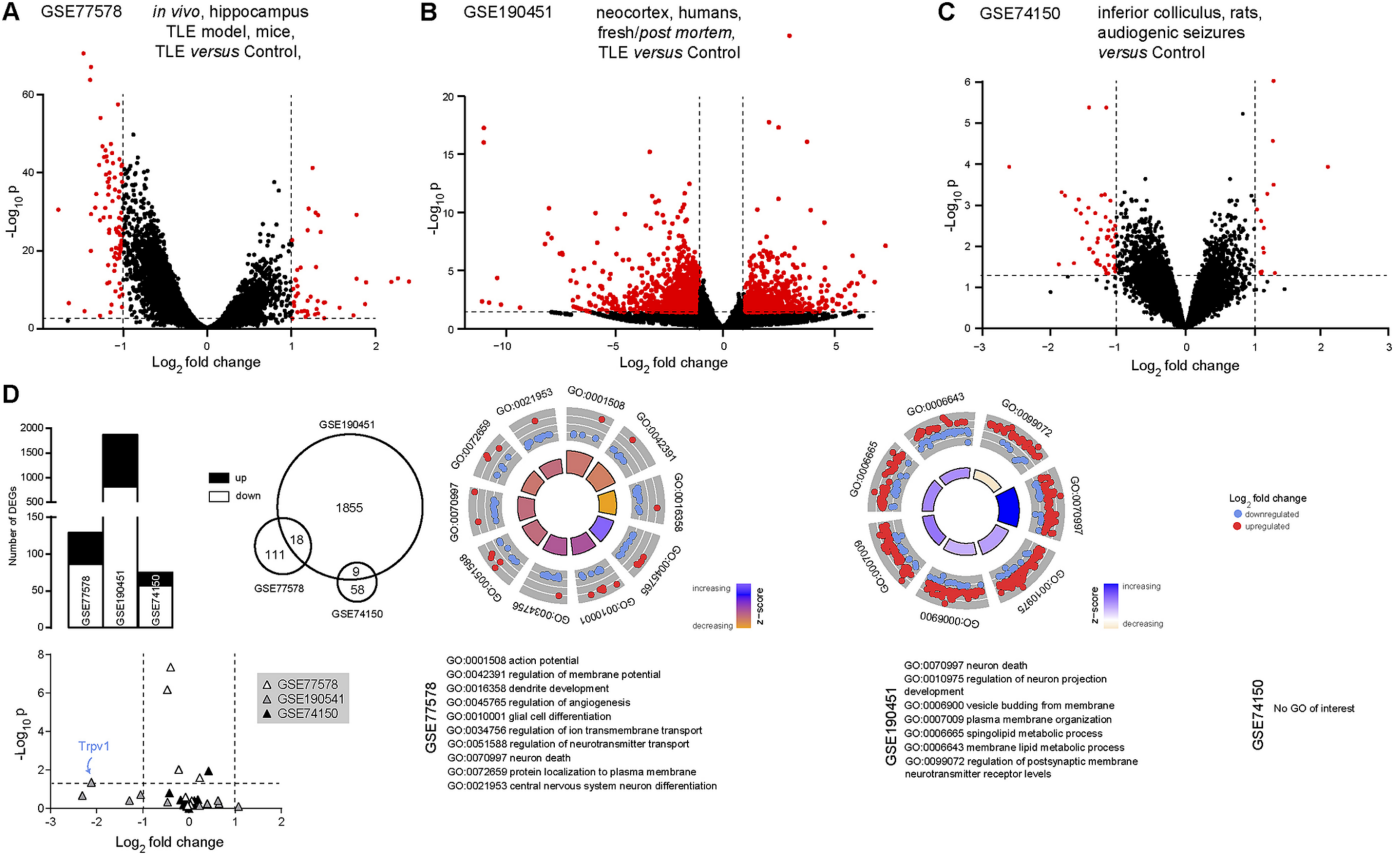
*TRPV1* is significantly downregulated in temporal lobe epilepsy. **(A)** Volcano plot comparing transcript expression in control mice to mice with temporal lobe epilepsy (pilocarpine model), extracted from the GSE77578 dataset (Srivastava et al., 2018). **(B)** Volcano plot comparing transcript expression in tissue samples from control patients to fresh tissue samples extracted from the epileptogenic foci from patients with temporal lobe epilepsy [GSE190451 dataset (Chen et al., 2023)]. **(C)** Volcano plot comparing transcript expression in control rats to a genetic model of epilepsy [Wistar audiogenic rat, Damasceno et al., 2020]. Red dots indicate significantly up- or down-regulated transcripts. Horizontal bars represent the significant threshold, set at p = 0.05 (−log_10_(p) = 1.3). Vertical bars represent the threshold for significant differences in transcript expression, with either down- (<−1) or up- (>1) regulated transcripts. **(D)** Histograms (left), Venn diagram (middle) and gene ontology plots (right) showing small overlap between the significantly up- or down-regulated transcripts in the three datasets. On gene ontology plots, several biological processes are found dysregulated in both datasets, including neurophysiology and neuronal death. *TRPV1* (bottom, blue arrow) is significantly down-regulated in epilepsy (human, GSE190451). GO, gene ontology; TLE, temporal lobe epilepsy.

Our next step was to investigate the eCB system with regard to temporal lobe epilepsy, in light of extensive evidence of the links between the two (Alger, 2004; Kwan Cheung et al., 2019; Bilbao and Spanagel, 2022; Martínez-Aguirre et al., 2022; Sugaya and Kano, 2022). Three datasets were thus investigated: GSE77578 (Srivastava et al., 2018), GSE190451 (Chen et al., 2023) and GSE74150 (Damasceno et al., 2020). In GSE77578, 56 hippocampal samples were compared from epileptic or control mice. Here, we only compared control mice to epileptic mice (temporal lobe epilepsy), excluding mice treated with an antiepileptic drug. We found 129 differentially expressed transcripts in such a model of temporal lobe epilepsy, with 43 transcripts up-regulated and 86 down-regulated (Figure 3A). In GSE190451, 6 neocortical samples were compared between patients with temporal lobe epilepsy (*n* = 3) and aged-matched non-epileptic controls (*n* = 3), an analysis that differs slightly from the published article, in which the authors analyzed a total of 8 samples (Chen et al., 2023). We found a total of 1873 differentially expressed transcripts in temporal lobe epilepsy, 833 up-regulated and 1,040 down-regulated (Figure 3B). In GSE74150, an animal model of genetic epilepsy was used (Wistar audiogenic rat). Here, we found 67 differentially expressed transcripts, 53 down-regulated and 14 up-regulated (Figure 3C). Up- and down-regulated transcripts in the three datasets presented some overlap (Figure 3D, Supplementary Table S3), showing that transcripts involved in, or responding to, epilepsy seem to be found across species and models. Several gene ontology enrichments (ORA) were detected in the first two datasets, ranging from regulation of neurophysiology to neuronal death. Interestingly, 37 biological process terms were found to be common across the two first datasets (Supplementary Table S4), such as synapse organization, neuron death, myelination and plasma membrane organization (Figure 3C). GSEA analyzes did not reveal any biological processes of interest (not shown). Out of our 13 transcripts of interest, *TRPV1* was found to be significantly down-regulated in the neocortex of patients with temporal lobe epilepsy (Figure 3D). These results confirm, once again, that *TRPV1* plays an important role in the pathophysiology of epilepsy, as observed previously (Jia et al., 2015; Saffarzadeh et al., 2015; Kong et al., 2019; Wang et al., 2019; Lazarini-Lopes et al., 2022).

### 3.4 The eCB system interactome

**Figure 4:**
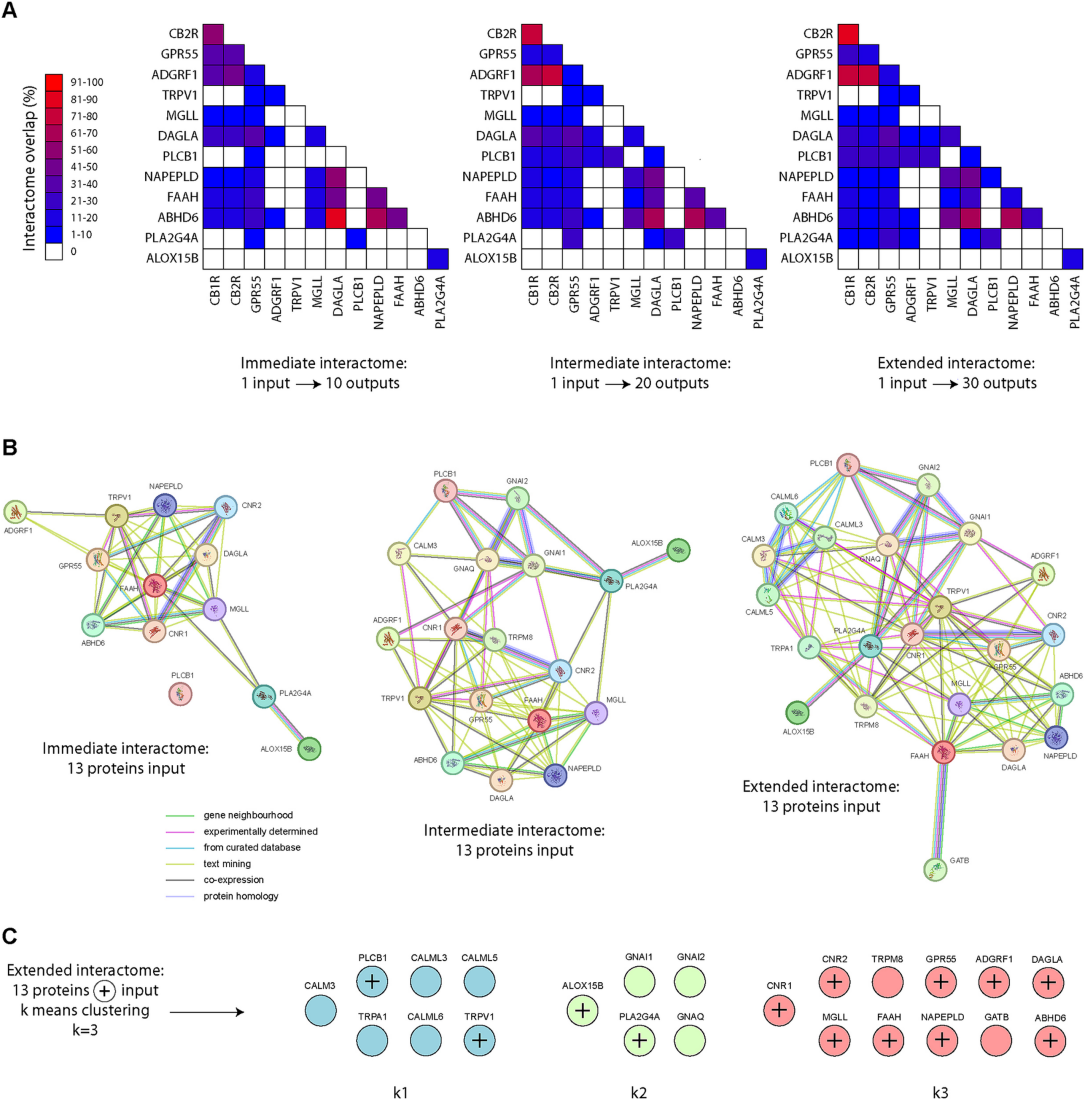
Endocannabinoid protein interactomes. **(A)** Pair-wise matrix comparisons of immediate, intermediate or extended protein interactomes overlap, using single proteins as input into the STRING database, from our list of 13 proteins of interest (based on the 13 transcripts of interest). Note the high interactome overlap with CB1R, CB2R, GPR55, ADGFR1 (GPR110) on one side and NAPE-PLD, DAGLA and ABHD6 on the other. The interactome of ALOX15B appears to not overlap with the other 12 proteins of interest, except PLA2G4A (14.3–18.2%). **(B)** Protein interactomes at an immediate, intermediate or extended level. **(C)** K means clustering (*k* = 3) was applied to identify segregation groups within the extended endocannabinoid protein interactome.

Using the STRING database (Szklarczyk et al., 2019), containing both known and predicted protein interactions, we first fed into the database single protein names (from our list of 13 transcripts/proteins). Analysis of the immediate interactomes revealed great disparities in terms of protein overlap in pair-wise comparisons (Figure 4A). Interestingly, great overlap was observed with CB1R, CB2R and ADGRF1 (GPR110). We also noted great protein overlap in the interactomes of DAGLA, NAPE-PLD and ABHD6. As we expanded the interactome searches, similar conclusions were reached (Figure 4A). Surprisingly, TRPV1 and ALOX15B presented the lowest overlap in all levels, whether immediate, intermediated or extended interactomes (Figure 4A). We next scanned the database using our 13 proteins of interest by bulk feeding all 13 proteins into the search equation. When examining the immediate interactome of these 13 proteins, only the same 13 proteins were returned (Figure 4B), providing no additional information of interest. However, when the interactome search was expanded to an intermediate level, new proteins appeared, including GNAI1, GNAI2, GNAQ, and TRMP8 (Figure 4B). When the search was expanded to another level (extended interactome), new protein interactions arose, such as CALM3, CALML3, CALML5, CALML6 and TRPA1 (Figure 4B). Our final step was to apply k means clustering using a fixed number of clusters (*k* = 3). Very surprisingly, 4 proteins clustered outside of the main cluster containing the proteins of interest: TRPV1, PLC1B, ALOX15B and PLA2G4A (Figure 4C). We also noted that TRPM8 and GATB clustered with the majority of our proteins of interest, via FAAH, acting as a link within the extended interactome (Figure 4C).

### 3.5 Paralogues of human CBRs

**Figure 5:**
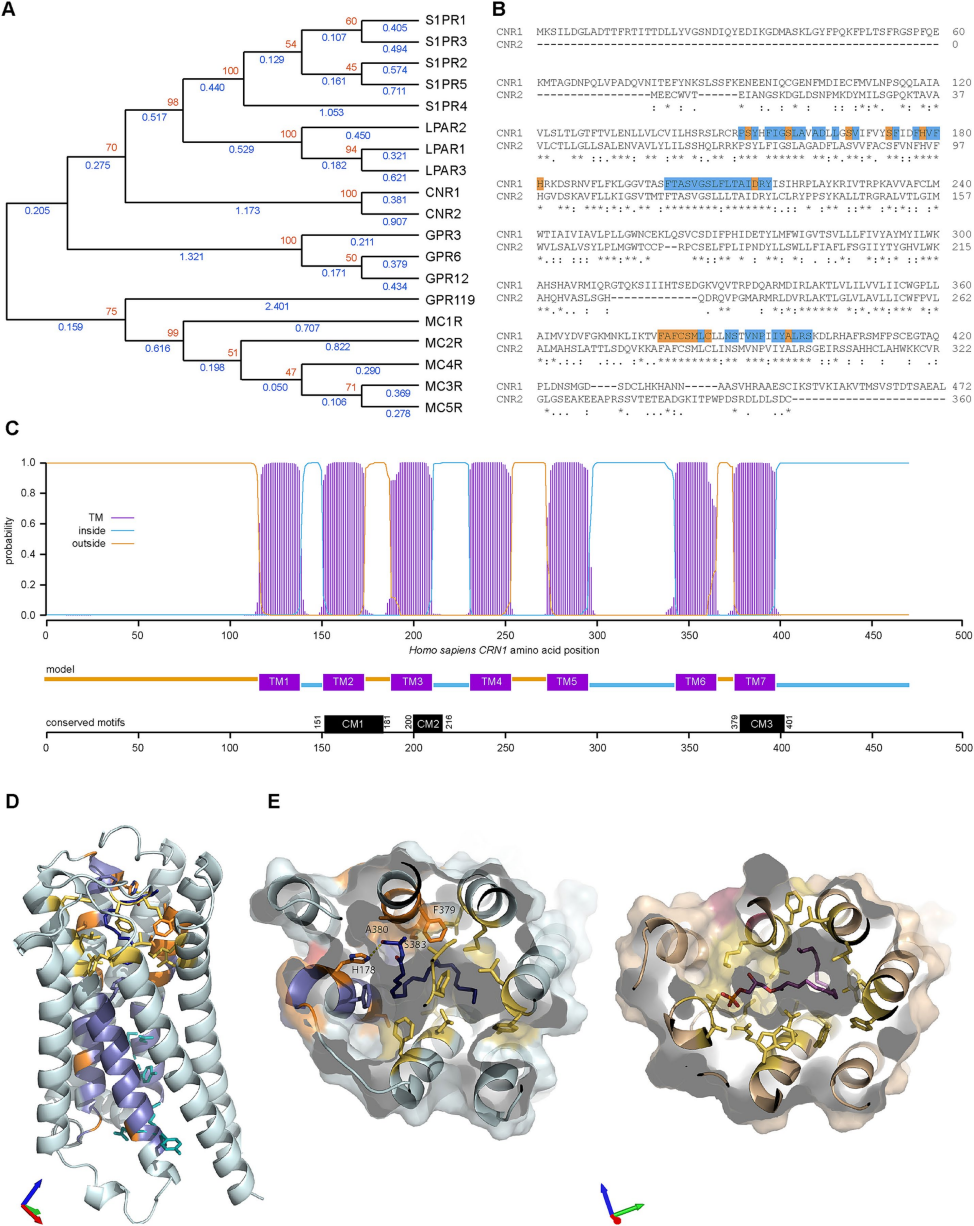
Paralogues of CNR1 in *Homo sapiens* and evolution of CB1R structure. **(A)** Phylogenic relationships of cannabinoid receptor 1 (CNR1) and the eighteen CNR1 paralogues listed in Genecards. The evolutionary history was inferred by using the Maximum Likelihood method and Jones-Taylor-Thornton matrix-based model (Jones et al., 1992). The tree with the highest log likelihood (−13679.67) is shown. The percentage of trees in which the associated taxa clustered together is shown next to the branches (orange, from 100 bootstraps). Initial trees for the heuristic search were obtained automatically by applying Neighbor-Join and BioNJ algorithms to a matrix of pairwise distances estimated using the JTT model, and then selecting the topology with superior log likelihood value. A discrete Gamma distribution was used to model evolutionary rate differences among sites [3 categories (+*G*, parameter = 1.3423)]. This analysis involved 19 amino acid sequences. There were a total of 504 positions in the final dataset. Blue values indicate branches length. **(B)** Clustal Omega alignment of *Homo sapiens* (Hs) CNR1 and CNR2. Runs (≥ 3 amino acids or longer runs with >80% identity) that are present in CNR1/2 but absent in other Hs GPCR paralogues are highlighted in blue with unique CNR amino acids (compared to S1PR and LPAR) in orange on CNR1 sequence. Three areas of interest are identified, conserved motif (CM) 1 (residues 151–181 in CNR1), CM2 (200–216) and CM3 (379–401). **(C)** Transmembrane Helices Hidden Markov Models scan of CNR1. CM1 is located in TM (transmembrane) 2, CM2 in TM3 and CM3 in TM7. **(D)** *Homo sapiens* cannabinoid receptor type 1 (PDB: 8GHV) bound to anandamide analog AMG315 (dark blue). Residues conserved in CNR, LPAR and S1PR clades are colored blue and those conserved in only the CNR clade are colored orange. Residues critical for receptor activation are shown as cyan sticks and additional, non-conserved residues in the anandamide binding pocket are colored yellow. Axes colored X red, Y green, Z blue. **(E)** Comparison of *Homo sapiens* cannabinoid receptor type 1 (left) and *Homo sapiens* lysophosphatidic acid receptor (right, PDB: 7TD1) binding pockets. Axes colored X red, Y green, Z blue. Color scheme as in **(D)** with lysophosphatidic acid show in magenta.

Our next step was to try to determine how CB1R (the protein produced from the *CNR1* gene) evolved within the human proteome. Using GeneCards (Stelzer et al., 2016) with *Homo sapiens* CB1R as input, 18 paralogues were returned. As expected, the closest paralogue was CNR2 (Figure 5A). CNR1 and CNR2 formed a distinct clade, with the next most closely related G-coupled protein receptors (GPCRs) being lysophosphatidic acid receptors (LPARs) and sphingosine-1-phosphate receptors (S1PRs). The furthest clade included melanocortin receptors 1–4 (MCRs) and GPR119. Inspection of the sequences revealed three conserved regions (Figure 5B), which could be divided into residues conserved within the clade containing CNR, LPAR and S1PR families (Figure 5B, blue) and residues only conserved within the CNR clade (Figure 5B, orange). Three conserved motifs (CMs) were identified: CM1, CM2 and CM3 are located within transmembrane (TM) 2, 3 and 7, respectively (Figure 5C). We then examined the location and function of these residues in the *Homo sapiens* CB1R structure bound to an anandamide analog (Krishna Kumar et al., 2023) and in the lysophosphatidic acid receptor structure bound to its native lysophosphatidic acid ligand (PDB: 7TD1) (Liu et al., 2022). The residues conserved within all three CNR, LPAR and S1PR families are largely found in the lower part of the receptor and include five of the six residues identified as being important in receptor activation (Figure 5D), as observed before (Hua et al., 2020). The ligand binding pocket in both receptors is formed by a large pocket of hydrophobic residues. Most of the residues conserved only within the CNR clade are located in this area and include some hydrophobic residues and H178 which forms a hydrogen bond with the terminal hydroxyl of the ligand (Figures 5D,E).

**Figure 6:**
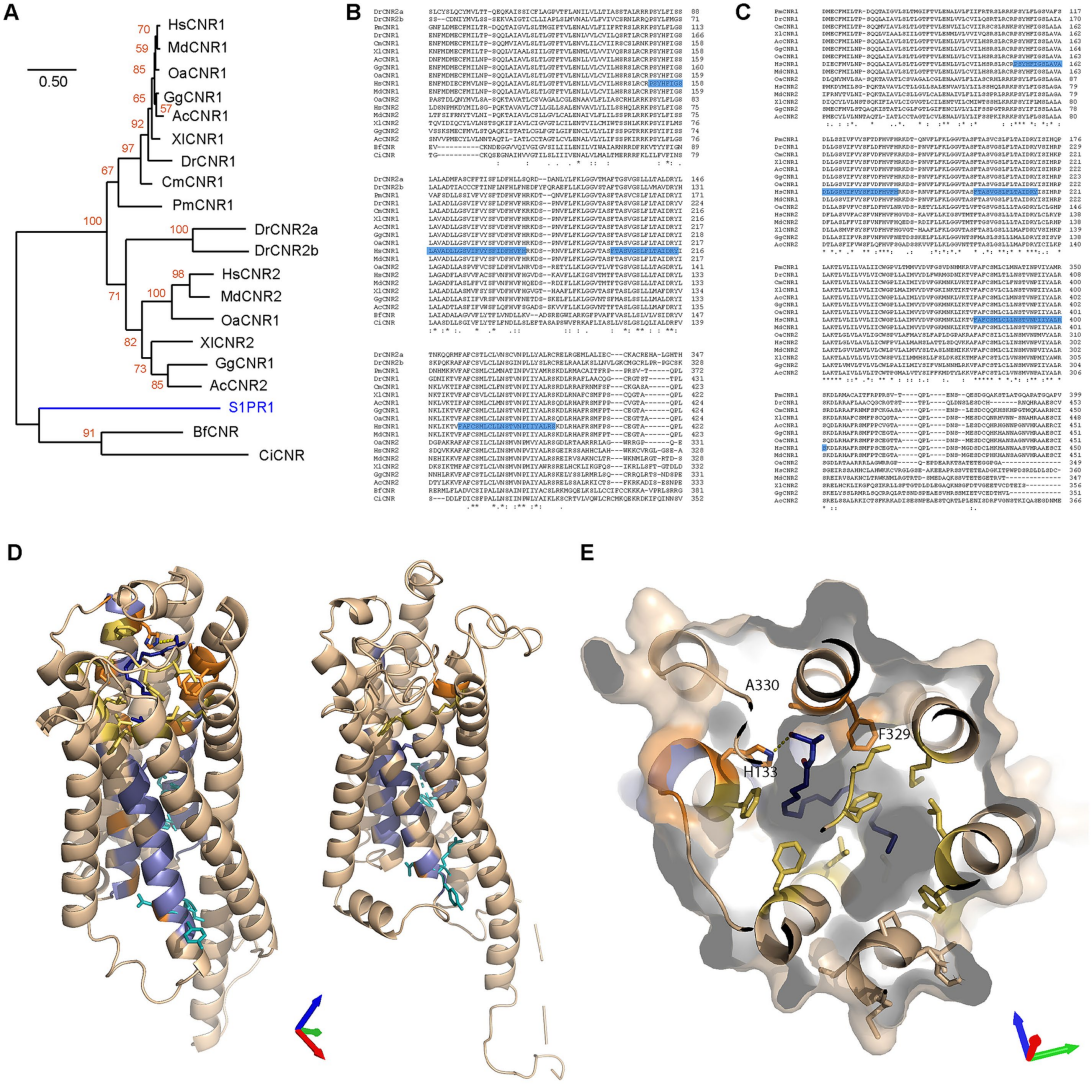
Evolutionary analysis of CNR1/CB1R in different taxa and structural comparisons. **(A)** Maximum Likelihood analysis using a neighbor joining method. Sequences aligned with Clustal W and phylogenetic analysis carried out using JTT + G with 100 bootstraps (orange values). Sphingosine-1-phosphate receptor 1 (S1PR1) was used to root the tree (blue branch). **(B)** Alignment of all CNR proteins. The three motifs identified earlier are highlighted in blue. Sequence not containing the conserved motifs were removed from the figure. **(C)** Alignment of CNR proteins identified in chordates (with the removal of Teleost CNR2). Sequence not containing the motifs removed from the figure. Blue highlighting are residues that were identified earlier as conserved (from CM1, CM2 and CM3). **(D)** Homology models of *Petromyzon marinus* CB1R superposed onto the anandamide analog AMG315 (dark blue) (left) and *Branchiostoma floridae* cannabinoid receptor of unknown type (right). Residues conserved in CNR, LPAR and S1PR clades are colored blue and those conserved in only the CNR clade are colored orange. Residues critical for receptor activation are shown as cyan sticks and additional, non-conserved residues in the anandamide binding pocket are colored yellow. Axes colored X red, Y green, Z blue. **(E)** *Petromyzon marinus* CB1R superposed to the anandamide analog binding pocket. Axes colored X red, Y green, Z blue. Color scheme as in **(D)**.

Out of 13 deuterostome species spanning from mammals to echinoderms (plus insects as protostomes), CB1R orthologues were found in placental mammals, marsupials, monotremes, birds, reptiles, amphibians, teleosts, elasmobranchs and Agnatha (Supplementary Table S5), but absent in cephalochordates, tunicates, echinoderms and insects. Furthermore, CB1R orthologues were also not found in plants, bacteria, Cnidaria and Porifera, except in Klebsiella (gram-negative bacteria) which presented 100% homology with the bird CB1R sequence (not shown), thus likely accounting for a database error in GenBank. When the same experiment was conducted with CB2R, we observed the presence of orthologues in placental mammals, marsupials, monotremes, birds, reptiles, amphibians and teleosts, the latter presenting two orthologues (Supplementary Table S5). Within the BLASTP database, unknown CBRs were found in cephalochordates and tunicates. These results suggest that the classical endocannabinoid receptors (CB1R and CB2R) are confined to a subgroup of deuterostomes, containing urochordates, cephalochordates and chordates, but absent from other deuterostomes, other animals, plants and prokaryotes, thus partly corroborating previous observations (McPartland et al., 2006; Elphick, 2012). Furthermore, it is suggested that a duplication event has occurred in teleosts since they diverged from other chordates. A further phylogenetic analysis revealed a clear division between CB1R and CB2R into two distinct clades (Figure 6A). We noted that most taxa presented copies of both proteins. There are two explanations for the lack of CNR2 paralogues in agnathans and elasmobranchs: (i) a duplication may have occurred since the divergence of other chordates and elasmobranchs or (ii) CNR2 paralogues were lost by both agnathans and elasmobranchs, the first scenario being by far the more likely explanation. Finally, we noted that the CBRs of Cephalochordates and Tunicates are very different, making it difficult to distinguish these two proteins from other related GPCRs. To address this, we performed multiple alignments with CB1R and CB2R protein sequences from the above-mentioned species. Our first alignment included cephalochordates and tunicates and led to great disruptions of the CMs (Figure 6B). The second alignment, which was performed without cephalochordates and tunicates, produced more defined, but yet not optimal, CMs (not shown). Our final alignment was performed without cephalochordates, tunicates and *Danio rerio*. This final approach produced robust identification of the three CMs (Figure 6C), thus corroborating the above results. Subsequently, we created homology models of the agnathan CB1R and cephalochordate unclassified CBR. Both the agnathan (*Petromyzon marinus*: ModelArchive ma-yvfgy) and cephalochordate (*Branchiostoma floridae*: ModelArchive ma-mi5fb) receptors contain the six residues important for receptor activation. However, the cephalochordate receptor lacks the majority of the residues involved in the AEA-binding pocket, including a histidine equivalent (H178) needed for hydrogen-bond formation with the terminal hydroxyl (Figures 6D,E).

### 3.6 Paralogues of human TRPVs

**Figure 7:**
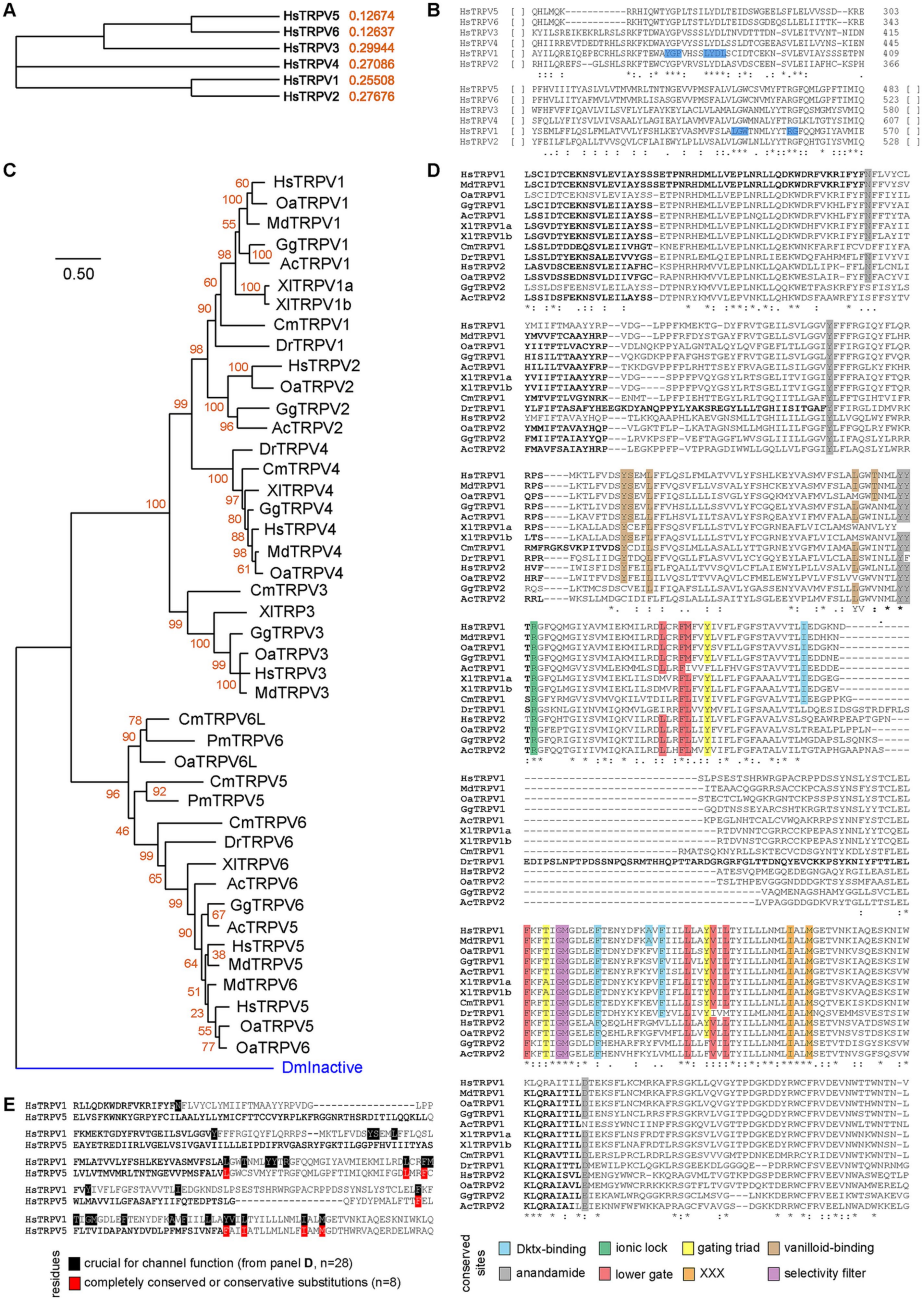
Phylogeny of TRPVs and conserved residues. **(A)** Simple phylogeny illustrating the relationships of the six TRPVs human paralogues. **(B)** Clustal alignment of the six *Homo sapiens* TRPVs. Two small fully conserved motif highlighted in blue. **(C)** Minimum Evolution Tree of Chordate TRPV family. Aligned using ClustalW, and ME tree (JTT + G) with 500 bootstraps. DmInactive (proteostome TRPV protein) is used to root the tree (blue branch). **(D)** Alignment of TRPV1 and TRPV2 showing conservation of sites. In each case, highlighted residues are either fully conserved or with conservative substitutions. **(E)** Comparison of TRPV1 and TRPV5, aligned using ClustalW.

Our final analyzes focused on TRPV1, as this is the second eCB receptor in the central nervous system (together with CB1R). Using similar approaches, we first determined the phylogeny of the six *Homo sapiens* paralogues, using TRPV1 as the reference sequence. We observed that TRPV1 and TRPV2 diverged recently, which arose from a duplication event (Figure 7A). Likewise, divergence of TPRV3, TRPV5 and TRPV6 arose earlier, with TPRV5 and TRPV6 being the most recent split (Figure 7A). Two small CMs were noted throughout those 6 different proteins, including Y375-G376-P377, L382-Y383-D384-L385, L547-G548-W549 and R557-G558 (Figure 7B). Using *Homo sapiens* TRPV1 sequence as bait, we found that TPRV5 or TRPV6 were present across the most diverse range of taxa (Supplementary Table S6). These receptors are present in Bilatera but absent from Porifera, the latter presenting weak protein homology. These observations suggest that the TRPV family is specific to the Bilatera. Furthermore, TPRV1 and TRPV2 resulted from later duplication events in the common ancestor of jawed vertebrates and amniotes, respectively (Figure 7C). In addition, our phylogenetic analysis highlighted that eCB-binding receptors (TRPV1 and TRPV2) form one clade, which includes taxa from elasmobranchs onwards, with a more recent duplication forming TRPV2, found in amniotes only (Figure 7C), as suggested above (Supplementary Table S6). Alignments of TPRV1 and TRPV2 from these species correctly identified conserved residues involved in channel gating and selectivity, as well as ligand binding [anandamide, vanilloid, double-knot toxin (DkTx), Figure 7D]. Conserved residues were often found throughout species, indicative of the importance of these residues for TRPVs function. Finally, out of these 28 functional residues in TRPV1, only 8 were conserved in TRPV5, whether fully conserved or present with conservative substitutions (Figure 7E). Taken together, these results pinpoint a divergence of eCB receptors between jawed and jawless vertebrates, around 450 million years ago during the late Ordovician. These observations are consistent with previous studies (Elphick et al., 2003; McPartland, 2004; McPartland et al., 2006).

**Figure 8:**
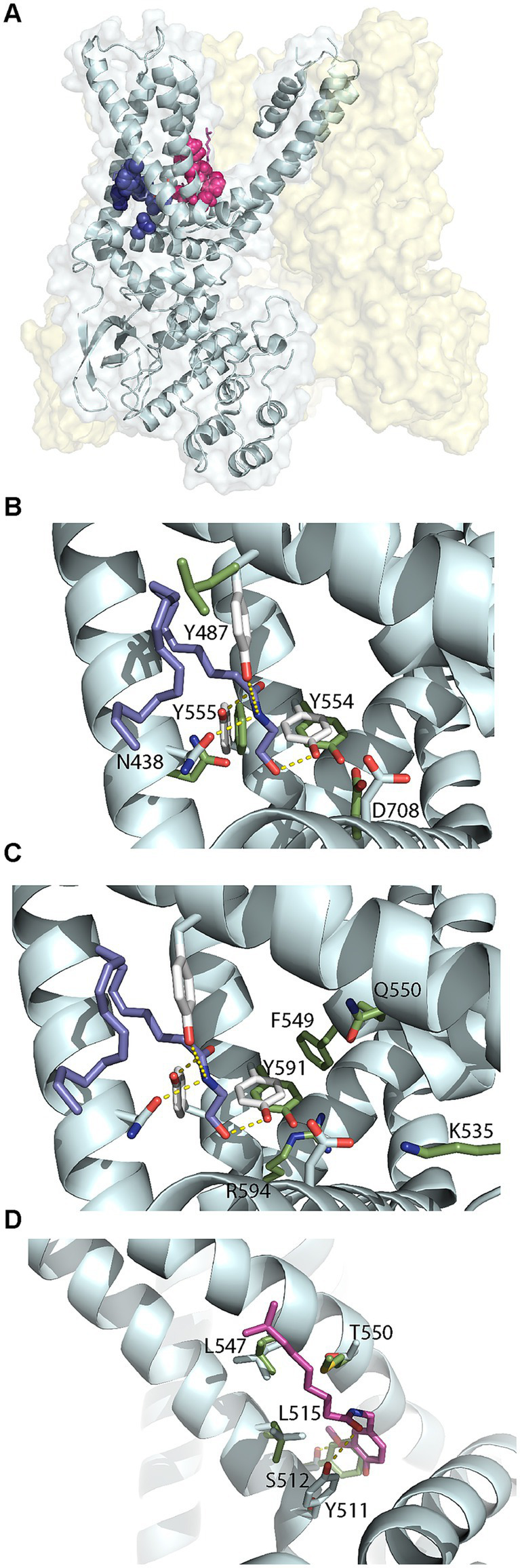
Structural comparisons of *Homo sapiens* TRPV1 and TRPV4. **(A)** *Homo sapiens* TRPV1 (PDB: 8GFA) showing one of four monomers in detail. Anandamide (AEA) tunnel residues shown in dark blue and vanilloid-binding pocket residues shown in pink. **(B)** Comparison of residues in the AEA tunnel for TRVP1 (white sticks with residue labels) and TRPV4 (green sticks, PDB: 8TD1). AEA is shown in blue sticks. **(C)** Comparison of residues in the AEA tunnel for TRVP1 (white sticks) and the 5′,6′-EET pocket for TRPV4 (green sticks with residue labels). AEA is shown in blue sticks. **(D)** Comparison of residues in the vanilloid-binding pocket for TRVP1 (white sticks with residue labels) and TRPV4 (green sticks). Capsaicin (natural phytochemical ligand) is shown in magenta.

We examined the structures of TRPV1 and TRPV4 to identify residues important for ligand binding. TRPV1 can be activated by endogenous ligands such as AEA and exogenous ligands such as capsaicin, the component of chili peppers which creates a sensation of burning (Lam et al., 2005). Structural studies have identified the capsaicin binding site, known as the vanilloid-binding pocket (Nadezhdin et al., 2021; Kwon et al., 2022). There are no experimentally determined structures of the TRPV1 receptor bound to AEA. However, using a model of human TRPV1 based on the experimental rat structure, molecular dynamics and docking studies have identified two possible binding sites for anandamide (Muller et al., 2020; Morales et al., 2022), represented in Figure 8A. One site was the vanilloid-binding pocket (VBP) but the preferred site was a tunnel (AEA tunnel) involving interactions with Y554, Y555, Y487, D708, and N438. We docked AEA into the AEA tunnel using the recently experimentally determined structure of human TRPV1 (PDB: 8GFA) (Neuberger et al., 2023). One of nine docking solutions had a similar orientation to that shown in Morales *et alia* (Morales et al., 2022), with Y555, N438 and Y554 forming hydrogen bonds with the amide group hydrogen and the terminal hydroxyl group oxygen (Figure 8B, ModelArchive ma-dol4y). The five interacting residues are well conserved across species in TRPV1 and TRPV2. AEA itself does not directly bind to TPRV4, however this receptor is activated by an AEA metabolite 5′,6′-epoxyeicosatrienoic acid (5′,6′-EET) (Watanabe et al., 2003). *Homo sapiens* TRPV4 (PDB: 8TD1) (Nadezhdin et al., 2021) is missing equivalents to TRPV1 Y847 and Y555 which stabilize AEA with hydrogen bonds in the AEA tunnel. Simulations have suggested that residues K535, F549, Q550, Y591 and R594 of TRPV4 form the 5′,6′-EET binding pocket (Berna-Erro et al., 2017), as displayed in Figure 8C. Of these, Y591 is equivalent to Y554 in TRPV1 which forms a hydrogen bond with the terminal hydroxyl of AEA, but the remaining pocket is very different. We also examined differences in the vanilloid-binding pocket by superimposing capsaicin from the rat (*Rattus norvegicus*) TRPV2 complex structure (PDB: 8SLY) (Gochman et al., 2023) onto the *Homo sapiens* TRPV1 receptor (PDB: 8GFA). Whilst some important hydrophobic residues are conserved in the equivalent pocket in TRPV4, there are no equivalent residues for the hydrogen-bonding Y511 and T550 which were previously observed to be involved in propagating ligand-activated conformational changes (Figure 8D) (Kwon et al., 2022).

## 4 Discussion

The location of CB1Rs is restricted to pre-synaptic terminals of both excitatory and inhibitory neurons (Mátyás et al., 2008). The physiological roles of CB1R are to mediate mood (Sbarski and Akirav, 2018), memory (Borgan et al., 2019a), stress (Arévalo et al., 2001; Barna et al., 2004) and locomotion (Han et al., 2023). These functions were reviewed in 2020 (Cristino et al., 2020). Activation of CB1Rs induces synaptic plasticity via long-term depression (Wu et al., 2015). Indeed, following synaptic activation, endogenous endocannabinoids (AEA and 2-AG) will be released from the post-synaptic element to then diffuse to the pre-synaptic element, where both molecules will act as agonists at CB1 receptors. Such activation of CB1 receptors will reduce neurotransmitter release, ending the cycle of synapse activation. This mechanism is often referred to as endocannabinoid-mediated negative feedback. Interestingly, CB1Rs were found to be expressed in the nucleus accumbens of vervet monkeys (Kucera et al., 2018), thus highlighting the role of these receptors in the reward circuitry (Méndez-Díaz et al., 2019; Braunscheidel et al., 2022; Ceccarini et al., 2022; Han et al., 2023). Dysfunction of CB1Rs has been linked to a wide array of neurological disorders such as Parkinson’s Disease, Alzheimer’s Disease, Huntington Disease and multiple sclerosis [reviewed in Cristino et al. (2020)].

In contrast to CB1R, TRPV1 is expressed in both presynaptic (Tominaga et al., 1998; Medvedeva et al., 2008) and postsynaptic (Bae et al., 2004; Grueter et al., 2010) elements. The physiological roles of TRPV1 intertwined those of CB1R, such as memory and locomotion, but also other functions, such as food intake (Cristino et al., 2020). Extensive evidence suggests that TRPV1 can be linked to epilepsy (Jia et al., 2015; Saffarzadeh et al., 2015; Kong et al., 2019; Wang et al., 2019; Lazarini-Lopes et al., 2022), although causal determination remains unclear. Indeed, in naïve rodents, chronic exposure to the TRPV1 agonist capsaicin induces spontaneous seizures (Jia et al., 2015). On the other hand, pre-treatment with the TRPV1 antagonist capsazepine decreased seizure severity and mortality in pentylenetetrazol-induced epilepsy (GABA_A_ antagonist) (Jia et al., 2015). Another study has observed altered electrophysiological properties in microglial cells following capsaicin, which was hypothesized as driving hyperthermia-induced seizures (Kong et al., 2019), although direct experimental confirmation has not been performed. Increased expression of TRPV1 was also observed after the successful induction of temporal lope epilepsy through kindling (audiogenic epilepsy) in rats (Lazarini-Lopes et al., 2022). Similar results were observed in another study using a model based on pilocarpine exposure (Saffarzadeh et al., 2015). Another elegant confirmation of the link between epilepsy and TRPV1 was observed in animals lacking TRPV1 (knock-out). Indeed, compared to wild-type mice, mice lacking TRPV1 presented decreased seizure latency, duration and severity (Wang et al., 2019). In agreement with these observations, our current transcriptomic results report a downregulation of TPRV1 in temporal lobe epilepsy. Indeed, we found a significant downregulation of TRPV1 mRNA in the neocortex of patients with temporal lobe epilepsy compared to control tissues. As mentioned above, preclinical studies have shown that agonism of TRPV1, using capsaicin, can induce tonic–clonic seizures, while TPRV1 antagonism reduced mortality in animals in which seizures were induced (Jia et al., 2015). These results were also observed in knock-out animals for TRPV1 (Jia et al., 2015). However, contrasting results were observed in a 2020 study, in which capsaicin inhibited epileptiform activities in the prefrontal cortex (Pasierski and Szulczyk, 2020). The same authors also witnessed decreased neuronal excitation, thus explaining the above-mentioned effects (Pasierski and Szulczyk, 2020), which are mediated by decreased sodium currents. These were not isolated results, as other studies have also concluded that capsaicin possesses anti-convulsive properties in rats (Abdel-Salam et al., 2020) and mice (Lee et al., 2011; Pezzoli et al., 2014). Interestingly, hippocampal expression of CB1Rs was also found to be significantly decreased in patients suffering from epilepsy (Ludányi et al., 2008). Thus, in light of these previous results and those from the present study, TRPV1 could be of interest for developing new therapeutics for patients with refractory epilepsy.

The role of the eCB system in neurodevelopmental disorders has been described previously. Indeed, many studies observed a direct relationship between altered CB1R availability in schizophrenia (Dean et al., 2001; Zavitsanou et al., 2004; Newell et al., 2006; Eggan et al., 2008; Urigüen et al., 2009; Wong et al., 2010; Dalton et al., 2011; Jenko et al., 2012; Ceccarini et al., 2013; Ranganathan et al., 2016; Muguruza et al., 2019; Bloch Priel et al., 2023) or during psychosis (Borgan et al., 2019b, 2021), which is also corroborated by animal models of schizophrenia (Szűcs et al., 2016; Stark et al., 2022). Furthermore, a link between THC exposure and schizophrenia has been evidenced in humans (Hjorthøj et al., 2021) and animal models (Rodríguez et al., 2017). Compared to control patients, levels of AEA are increased in the blood (De Marchi et al., 2003; Ibarra-Lecue et al., 2022) and cerebrospinal fluid (Giuffrida et al., 2004; Leweke et al., 2007; Minichino et al., 2019) in patients with schizophrenia. In addition, exposure to THC alters AEA levels (Leweke et al., 2007; Seillier et al., 2020; Ibarra-Lecue et al., 2022). Furthermore, lower brain FAAH availability was observed in adolescent chronic cannabis users (Jacobson et al., 2021). Targeting FAAH has proven an efficient treatment to decrease cannabis use and withdrawal (D’Souza et al., 2019). During psychosis, increased levels of DAGL and NAPE are reported in mononuclear cells (peripheral blood) compared to control patients (Bioque et al., 2013), two enzymes controlling endocannabinoid production. In addition, such a study also reported increased FAAH levels during psychosis (Bioque et al., 2013). Altogether, these studies provide ample evidence for a link between the eCB system and neurodevelopmental disorders. Recent studies reviewed the relationship of the eCB in several neurological pathologies such as depression (Gallego-Landin et al., 2021), schizophrenia (Garani et al., 2021), autism (Su et al., 2021) and substance use disorder (Navarro et al., 2022). In the current study, our transcriptomic analysis found that GPR55 is upregulated in the ventral tegmental area of adolescent mice following THC exposure, even after a long washout period (2 weeks). These results can be explained by the fact that THC possesses psychoactive effects (Ameri et al., 1999) and that the ventral tegmental area is known to respond to THC (French et al., 1997; Lupica et al., 2004; Ostlund et al., 2022). However, what remains surprising is the fact that GPR55, and not CB1R, is upregulated after THC exposure, in light of two studies demonstrating loss of CB1R-dependent plasticity after chronic THC exposure (Friend et al., 2017; Ostlund et al., 2022). The answer appears to lie within the capacity of GPR55 to respond to THC (Ryberg et al., 2007; Lauckner et al., 2008), which has been the reason why GPR55 is classed as an endocannabinoid receptor. In fact, THC, AEA, methanandamide (stable metabolic analog of AEA) and JWH015 (cannabinoid agonist) all seem to elicit intracellular responses in GPR55-transfected HEK cells (Lauckner et al., 2008), thus proving a link between GPR55 and THC. Furthermore, CBD is also known to abolish GPR55-mediated synaptic events (Sylantyev et al., 2013), highlighting again a strong interaction between GPR55 and the eCB system.

The two phytocannabinoids THC and CBD are known to bind to CB1R (Jakowiecki and Filipek, 2016; Hua et al., 2017; Chung et al., 2019), since the only chemical difference between these two compounds is a bond disconnection in CBD (Shao et al., 2016). Functionally, CBD has a low affinity for the CB1R (McPartland et al., 2007) and is described as an antagonist at CB1R (Thomas et al., 2007). However, evidence suggests that CBD rather behaves as a negative allosteric modulator of THC (Laprairie et al., 2015; Chung et al., 2019). Interestingly, CBD also binds to TRPV1-4 channels, as observed recently (Costa et al., 2004; Qin et al., 2008; Iannotti et al., 2014; Muller et al., 2019; Starkus et al., 2019; Galaj et al., 2020; Etemad et al., 2022; Landucci et al., 2022). These observations were also made outside of the central nervous system (De Petrocellis et al., 2012; Nabissi et al., 2013; de la Harpe et al., 2022).

We also observed that the amino acid sequence of TRPV1 in *Danio rerio* is missing Y554 and Y555, which have been shown to allow AEA binding (Muller et al., 2020). Instead, TRPV1 in zebrafish possesses A554 and A555. The switch from polar amino acids (Tyr-Tyr) to non-polar amino acids (Ala-Ala) in the TRPV1 orthologue of zebrafish does not seem to impact heat detection, which is the main function of TRPV1 (Zheng and Wen, 2019). Despite these amino acids substitutions, *in vitro* and *in vivo* observations confirmed that TRPV1 is triggered above 25°C in zebrafish (Gau et al., 2013). In contrast, heat activation of TRPV1 in mammals occurs above 42°C (Caterina et al., 1997), which could be explained by slightly different structural conformations following amino acid substitutions. Interestingly, TRPV3 seems to possess opposite heat-detection functions between humans and reptiles (*Xenopus tropicalis*), as observed before (Saito et al., 2011), with the receptor activated by cold temperatures (*≤* 16°C). Behaviorally, TRPA1 seems to be the receptor mediating avoidance of dangerous temperatures in frogs (Saito et al., 2022). Similar to our findings, Saito *et alia* also reported that, in vertebrates, TRPV channels form very distinct clades (Saito et al., 2011). Furthermore, TRPV1 and TRPA1 seem to have been co-expressed in an early vertebrate common ancestor (Saito et al., 2012).

The link between TRPV1 and heat can be experienced when eating hot chili peppers, which dose-dependently trigger receptor activation (Mohapatra and Nau, 2003; Voets et al., 2004; Neelands et al., 2005; Vetter et al., 2006; Vasquez et al., 2020; Matsuyama et al., 2021). THC can bind TRPV2-4, TRPA1 and TRPM8, but not TRPV1 (Muller et al., 2019). This seems very different from CBD, which has been shown to bind to TRPV1 (see above). What remains fascinating is the fact that TRPV channels are involved in sensory perception (chemical nociception, heat detection) in the peripheral nervous system, whilst TRPV1 in the central nervous system is involved in synaptic plasticity, neurite growth and signaling (Moran et al., 2004).

In terms of evolution, our results suggest that the eCB system appeared during the late Ordovician. McPartland and colleagues studied the evolution of all components of the eCB system in a study published in 2006 (McPartland et al., 2006). In their article, authors evidenced that TRPV1 and GPR55 are limited to mammals, whilst CB2R and DAGL were found amongst vertebrates (McPartland et al., 2006). Other components, such as MAGL, are found in chordates, while all eukaryotes express FAAH (McPartland et al., 2006). Thus, it can be safely hypothesized that the origins of the eCB appeared in a common eukaryotic ancestor. Such a study also suggested that ligand-metabolizing enzymes of the eCB system evolved prior to functional cannabinoid receptors (McPartland et al., 2006). In the absence of these receptors, such as CB1R, CB2R and TRPV1, endocannabinoids, acting as signaling molecules, such as AEA and 2-AG, might possess other functional properties (McPartland et al., 2006). A likely explanation is modulating physiological excitability in the nervous system (McPartland, 2004). 2-AG is expressed in insects and, apart from signaling properties, 2-AG might serve as a deterrent to predators, such as vertebrates, who do express functional receptors to 2-AG, such as TRPV1. This mechanism was likely driven by convergent evolution. Interestingly, this feeding-deterrent mechanism was also suggested for plants (McPartland et al., 2006), expressing endocannabinoid-like compounds but without eCB receptors. Intertwined evolution of receptors, enzymes and ligands of the eCB system was likely driven by convergence, divergence and parallel evolution (McPartland and Guy, 2004). To the best of our knowledge, pinpointing an exact timeframe for such mechanisms is currently impossible. However, appearance of the eCB in the late Ordovician seems to be the accepted consensus, and our current study further corroborates these previous suggestions.

Indeed, recent studies have observed brain-like structures in fossils from the Cambrian-Ordovician eras (Dong et al., 2022; Ortega-Hernández et al., 2022; Pates et al., 2022), which seem to support the idea of an earlier origin of the nervous system, likely occurring before the appearance of annelids (Parry and Caron, 2019). This pinpoints to the nervous system originating just before 500 million years ago, with the eCB system likely appearing 50 million years after the former. Further evidence has been gathered in invertebrates (annelids and insects), in which observations of eCB-like receptors were discovered (Stefano et al., 1997; Elphick, 1998, 2012; McPartland et al., 2006). Some components of the eCB system have been found in species much older than annelids. Indeed, FAAH, which is responsible for metabolism of AEA, has been identified in the moss *Physcomitrella patens*, with a high catabolic activity for AEA (Haq and Kilaru, 2020). Furthermore, 9 paralogues were identified in this species, with some closely related to rat FAAH, while others closely related to plant FAAH (Haq and Kilaru, 2020). It is now accepted that the nervous system appeared in bilaterians ancestors. Recovered fossils suggest that the nervous system was already present 540 million years ago (McPartland and Guy, 2004; Budd, 2013; Heger et al., 2020). Furthermore, several studies confirmed the presence of a nervous system in bilaterians (Adey, 1951; Holland et al., 2013; Yang et al., 2013, 2016; Martín-Durán et al., 2018), sponges (Leys, 2015; Musser et al., 2021; Yañez-Guerra et al., 2022) or ctenophores (Jékely et al., 2015; Moroz, 2015; Burkhardt et al., 2023). Interestingly, the oldest bilaterians fossils recovered so far range between 540 and 585 million years (Martin et al., 2000; Pecoits et al., 2012; Chen et al., 2019; Evans et al., 2020). Altogether, it can be safely assumed that the nervous system appeared before such a timeframe.

To conclude, we provide here new evidence that GPR55 is upregulated in the ventral tegmental area of mice following THC exposure and that TPRV1 is downregulated in the epileptogenic focus of patients with temporal lobe epilepsy. These results further support previous observations linking the eCB system to epilepsy, cannabis and neurodevelopmental disorders. We also report here an updated analysis of the evolution of CB1R and TRPV1, two major actors in the eCB system.

## Data availability statement

The datasets presented in this study can be found in online repositories. The names of the repository/repositories and accession number(s) can be found in the article/Supplementary material.

## Author contributions

AC: Data curation, Formal analysis, Investigation, Methodology, Software, Validation, Visualization, Writing – review & editing. MW: Data curation, Formal analysis, Investigation, Methodology, Software, Validation, Visualization, Writing – review & editing. KM: Formal analysis, Investigation, Writing – review & editing. MDM: Conceptualization, Data curation, Formal analysis, Funding acquisition, Investigation, Methodology, Project administration, Resources, Software, Software, Validation, Visualization, Writing – original draft, Writing – review & editing.

## Footnotes

^1^ https://cran.r-project.org

^2^ https://blast.ncbi.nlm.nih.gov/Blast.cgi?PAGE=Proteins

^3^ https://www.ebi.ac.uk/Tools/msa/clustalo/

^4^ https://services.healthtech.dtu.dk/services/TMHMM-2.0/

^5^ https://string-db.org/
