# Supplementary material for: In silico analyses of the involvement of GPR55, CB1R and TRPV1: response to THC, contribution to temporal lobe epilepsy, structural modeling and updated evolution
Source: Front Neuroinform. 2024 Feb 7;18:1294939. doi: 10.3389/fninf.2024.1294939 (PMC10894036; doi:10.3389/fninf.2024.1294939)
Supplement: Supplementary file 1 [file Data_Sheet_1.docx]

**Supplementary Material**

***Supplementary Table S1. Gene Expression Omnibus (GEO) datasets used in the present study.*** *GSE: GEO series experiment.* ***Table 1*** *contains detailed information.*

| Dataset | Accession link | Publication |
| --- | --- | --- |
| GSE80655 | https://www.ncbi.nlm.nih.gov/geo/query/acc.cgi?acc=GSE80655 | (Ramaker et al., 2017) |
| GSE189821 | https://www.ncbi.nlm.nih.gov/geo/query/acc.cgi?acc=GSE189821 | (Zuo et al., 2022) |
| GSE116813 | https://www.ncbi.nlm.nih.gov/geo/query/acc.cgi?acc=GSE116813 | (Jouroukhin et al., 2019) |
| GSE70689 | https://www.ncbi.nlm.nih.gov/geo/query/acc.cgi?acc=GSE70689 | (Juknat et al., 2013) |
| GSE77578 | https://www.ncbi.nlm.nih.gov/geo/query/acc.cgi?acc=GSE77578 | (Srivastava et al., 2018) |
| GSE190451 | https://www.ncbi.nlm.nih.gov/geo/query/acc.cgi?acc=GSE190451 | (Chen et al., 2023) |
| GSE74150 | https://www.ncbi.nlm.nih.gov/geo/query/acc.cgi?acc=GSE74150 | (Damasceno et al., 2020) |

***Supplementary Table S2****.* ***Genes of interest in the present study.*** *Thirteen* *genes were selected based on known actors of the eCB system.*

| **Gene** | **Full** **name** | **Function** |
| --- | --- | --- |
| CNR1 | cannabinoid receptor 1 | receptor |
| CNR2 | cannabinoid receptor 2 | receptor |
| TRPV1 | transient receptor potential cation channel, subfamily V, member 1 | receptor |
| GPR55 | G protein-coupled receptor 55 | receptor |
| GPR110 | G protein-coupled receptor 110 | receptor |
| MGLL | monoglyceride lipase | enzyme |
| DAGLA | diacylglycerol lipase, alpha | enzyme |
| PLCB1 | phospholipase C beta 1 | enzyme |
| NAPE-PLD | N-acyl phosphatidylethanolamine phospholipase D | enzyme |
| FAAH | fatty acid amide hydrolase | enzyme |
| ABHD6 | abhydrolase domain containing | enzyme |
| PLA2G4A | phospholipase A2 group 4A | enzyme |
| Alox8 | arachidonate 8-lipoxygenase | enzyme |

***Supplementary Table S3. List of differentially expressed transcripts found to be similar between GSE77578, GSE190451 and GSE74150.***

| **GSE77578 and GSE190451** | **GSE77578 and GSE74150** | **GSE190451and GSE74150** |
| --- | --- | --- |
| Aak1, Anxa2, Bcl11a, C1qb, Cd9, Cyba, Enpp2, Gfap, Ina, Kcnd2, Ly86, Ntrk2, Ptk2b, R100a6, Rerpinf1, Rorl1, Rparc, Rtrbp | *nil* | Eno4, Fcer1g, Itm2a, Krtcap2, Lair1, P2ry12, Pam16, Trem2, Tyrobp |

***Supplementary Table S4****.* ***Gene Ontology (GO) enrichments found in both GSE77578 and GSE190451.*** *Enrichment values represent the number of differentially expressed genes in each GO term over the total number of genes found differentially expressed. Here, genes not associated with any GO term were excluded from such calculations.*

| **Terms** | **Description** | **GSE77578 enrichment** | **GSE190451 enrichment** |
| --- | --- | --- | --- |
| GO:0006898 | receptor-mediated endocytosis | 6/115 | 41/1333 |
| GO:0007009 | plasma membrane organization | 5/115 | 19/1333 |
| GO:0007015 | actin filament organization | 7/115 | 45/1333 |
| GO:0007272 | ensheathment of neurons | 5/115 | 29/1333 |
| GO:0008366 | axon ensheathment | 5/115 | 29/1333 |
| GO:0010001 | glial cell differentiation | 8/115 | 41/1333 |
| GO:0010721 | negative regulation of cell development | 10/115 | 50/1333 |
| GO:0010976 | positive regulation of neuron projection development | 11/115 | 36/1333 |
| GO:0010977 | negative regulation of neuron projection development | 8/115 | 24/1333 |
| GO:0014013 | regulation of gliogenesis | 6/115 | 21/1333 |
| GO:0021782 | glial cell development | 4/115 | 26/1333 |
| GO:0022407 | regulation of cell-cell adhesion | 7/115 | 46/1333 |
| GO:0030100 | regulation of endocytosis | 5/115 | 30/1333 |
| GO:0030900 | forebrain development | 7/115 | 49/1333 |
| GO:0031345 | negative regulation of cell projection organization | 8/115 | 25/1333 |
| GO:0031346 | positive regulation of cell projection organization | 13/115 | 53/1333 |
| GO:0034063 | stress granule assembly | 2/115 | 6/1333 |
| GO:0042063 | gliogenesis | 10/115 | 53/1333 |
| GO:0042552 | myelination | 5/115 | 29/1333 |
| GO:0043112 | receptor metabolic process | 7/115 | 27/1333 |
| GO:0043523 | regulation of neuron apoptotic process | 6/115 | 28/1333 |
| GO:0045665 | negative regulation of neuron differentiation | 8/115 | 32/1333 |
| GO:0048259 | regulation of receptor-mediated endocytosis | 5/115 | 18/1333 |
| GO:0048709 | oligodendrocyte differentiation | 4/115 | 24/1333 |
| GO:0050768 | negative regulation of neurogenesis | 10/115 | 46/1333 |
| GO:0050808 | synapse organization | 9/115 | 47/1333 |
| GO:0051017 | actin filament bundle assembly | 5/115 | 22/1333 |
| GO:0051402 | neuron apoptotic process | 7/115 | 32/1333 |
| GO:0051961 | negative regulation of nervous system development | 11/115 | 48/1333 |
| GO:0061572 | actin filament bundle organization | 5/115 | 22/1333 |
| GO:0070997 | neuron death | 10/115 | 51/1333 |
| GO:1901214 | regulation of neuron death | 9/115 | 45/1333 |
| GO:1901215 | negative regulation of neuron death | 8/115 | 27/1333 |
| GO:1902903 | regulation of supramolecular fiber organization | 8/115 | 42/1333 |
| GO:1905517 | macrophage migration | 3/115 | 11/1333 |
| GO:1905521 | regulation of macrophage migration | 3/115 | 10/1333 |
| GO:1990778 | protein localization to cell periphery | 8/115 | 42/1333 |

***Supplementary Table S5***. ***Numbers of CBR protein orthologs in various deuterostomes (and insects (protostomes)).*** *Search carried out with BLASTP using CB1R from Homo sapiens as bait.*

| **Taxon** | **Species** | **CB1R orthologs** | **CB2R orthologs** | **unknown CNR** |
| --- | --- | --- | --- | --- |
| Placental mammals | *Homo sapiens* | 1 | 1 | 0 |
| Marsupials | *Monodelphis domestica* | 1 | 1 | 0 |
| Monotremes | *Ornithorhyncus anatinus* | 1 | 1 | 0 |
| Birds | *Gallus gallus* | 1 | 1 | 0 |
| Reptiles | *Anolis carolinensis* | 1 | 1 | 0 |
| Amphibians | *Xenopus laevis* | 1 | 1 | 0 |
| Teleosts | *Danio rerio* | 1 | 2 | 0 |
| Elasmobranchs | *Callorhincus milli* | 1 | 0 | 0 |
| Agnatha | *Petromyzon marinus* | 1 | 0 | 0 |
| Cephalochordates | *Branchiostoma floridae* | 0 | 0 | 1 |
| Tunicates | *Ciona intestinalis* | 0 | 0 | 1 |
| Echinoderms | *Strongylocentrotus purpuratus* | 0 | 0 | 0 |
| Insects | *Drosophila melanogaster* | 0 | 0 | 0 |

***Supplementary Table S6*** ***Numbers of TRPV protein paralogues by taxa.*** *Search carried out with BLAST within the GenBank database using TRPV1 from Homo sapiens as bait. Blue cells indicate paralogues outside of Chordata that are noticeably more divergent and fit outside of the core phylogenetic tree. Those represented by * are shortened versions and are not included in later phylogenetic reconstruction. The paralogue represented by † appears to be present (though not annotated) but its sequence is poor and is thus also excluded from phylogenetic reconstruction.*

| Taxon | Species | TRPV1 | TRPV2 | TRPV3 | TRPV4 | TRPV5/6 |
| --- | --- | --- | --- | --- | --- | --- |
| Placental mammals | *Homo sapiens* | 1 | 1 | 1 | 1 | 2 |
| Marsupials | *Monodelphis domestica* | 1 | 0**^†^** | 1 | 1 | 2 |
| Monotremes | *Ornithorhyncus anatinus* | 1 | 1 | 1 | 1 | 3 |
| Birds | *Gallus gallus* | 1 | 1 | 1 | 1 | 1 |
| Reptiles | *Anolis carolinensis* | 1 | 1 | 1* | 0 | 2 |
| Amphibians | *Xenopus laevis* | 2 | 0 | 1 | 1 | 1 |
| Teleosts | *Danio rerio* | 1 | 0 | 0 | 1 | 1 |
| Elasmobranchs | *Callorhincus milli* | 1 | 0 | 0 | 0 | 3 |
| Agnatha | *Petromyzon marinus* | 0 | 0 | 0 | 1* | 2 |
| Cephalochordates | *Branchiostoma floridae* | 0 | 0 | 0 | 0 | 2 |
| Tunicates | *Ciona intestinalis* | 0 | 0 | 0 | 0 | 2 |
| Echinoderms | *Strongylocentrotus purpuratus* | 0 | 0 | 0 | 0 | 3 |
| Insects | *Drosophila melanogaster* | 0 | 0 | 0 | 0 | 2 |

***Supplementary Material M1***. *Spreadsheet detailing our 7 DEG analyses. GSE codes are given for each sheet. Yellow highlighting indicates comparisons. padj: adjusted p value. Other abbreviations are detailed within the main manuscript or in figure legends.*

References for Supplementary materials

Chen, Z.-P., Wang, S., Zhao, X., Fang, W., Wang, Z., Ye, H., et al. (2023). Lipid-accumulated reactive astrocytes promote disease progression in epilepsy. *Nat. Neurosci.* 26, 542–554. doi: 10.1038/s41593-023-01288-6.

Damasceno, S., Gómez-Nieto, R., Garcia-Cairasco, N., Herrero-Turrión, M. J., Marín, F., and Lopéz, D. E. (2020). Top Common Differentially Expressed Genes in the Epileptogenic Nucleus of Two Strains of Rodents Susceptible to Audiogenic Seizures: WAR and GASH/Sal. *Front. Neurol.* 11, 33. doi: 10.3389/fneur.2020.00033.

Jouroukhin, Y., Zhu, X., Shevelkin, A. V., Hasegawa, Y., Abazyan, B., Saito, A., et al. (2019). Adolescent Δ9-Tetrahydrocannabinol Exposure and Astrocyte-Specific Genetic Vulnerability Converge on Nuclear Factor-κB-Cyclooxygenase-2 Signaling to Impair Memory in Adulthood. *Biol. Psychiatry* 85, 891–903. doi: 10.1016/j.biopsych.2018.07.024.

Juknat, A., Pietr, M., Kozela, E., Rimmerman, N., Levy, R., Gao, F., et al. (2013). Microarray and pathway analysis reveal distinct mechanisms underlying cannabinoid-mediated modulation of LPS-induced activation of BV-2 microglial cells. *PloS One* 8, e61462. doi: 10.1371/journal.pone.0061462.

Ramaker, R. C., Bowling, K. M., Lasseigne, B. N., Hagenauer, M. H., Hardigan, A. A., Davis, N. S., et al. (2017). Post-mortem molecular profiling of three psychiatric disorders. *Genome Med.* 9, 72. doi: 10.1186/s13073-017-0458-5.

Srivastava, P. K., van Eyll, J., Godard, P., Mazzuferi, M., Delahaye-Duriez, A., Van Steenwinckel, J., et al. (2018). A systems-level framework for drug discovery identifies Csf1R as an anti-epileptic drug target. *Nat. Commun.* 9, 3561. doi: 10.1038/s41467-018-06008-4.

Zuo, Y., Iemolo, A., Montilla-Perez, P., Li, H.-R., Yang, X., and Telese, F. (2022). Chronic adolescent exposure to cannabis in mice leads to sex-biased changes in gene expression networks across brain regions. *Neuropsychopharmacol. Off. Publ. Am. Coll. Neuropsychopharmacol.* 47, 2071–2080. doi: 10.1038/s41386-022-01413-2.

# Data Availability Statement

The datasets analyzed for this study are available open-access and can be found at the following Gene Expression Omnibus (GEO) links:
GSE80655 (<https://www.ncbi.nlm.nih.gov/geo/query/acc.cgi?acc=GSE80655>),
GSE189821 (<https://www.ncbi.nlm.nih.gov/geo/query/acc.cgi?acc=GSE189821>),
GSE116813 (<https://www.ncbi.nlm.nih.gov/geo/query/acc.cgi?acc=GSE116813>),
GSE70689 (<https://www.ncbi.nlm.nih.gov/geo/query/acc.cgi?acc=GSE70689>),
GSE77578 (<https://www.ncbi.nlm.nih.gov/geo/query/acc.cgi?acc=GSE77578>),
GSE190451 (<https://www.ncbi.nlm.nih.gov/geo/query/acc.cgi?acc=GSE190451>),
GSE74150 (<https://www.ncbi.nlm.nih.gov/geo/query/acc.cgi?acc=GSE74150>).

The following protein structures have been used, freely available at the Protein Data Bank (PDB) (<https://www.rcsb.org/>) with the following accession codes:
8GHV <https://www.rcsb.org/structure/8GHV>
7TD1 <https://www.rcsb.org/structure/7TD1>
8GFA <https://www.rcsb.org/structure/8GFA>
8T1D https://www.rcsb.org/structure/8T1D
8SLY <https://www.rcsb.org/structure/8SLY>

Homology models were created in Phyre^2^ (Kelley and Sternberg, 2009; Kelley et al., 2015), accessible at <http://www.sbg.bio.ic.ac.uk/~phyre2/html/page.cgi?id=index>. Docking of anandamide into *Homo sapiens* TRPV1 was performed with AutoDock Vina (Trott and Olson, 2010). All models were deposited in ModelArchive (<https://www.modelarchive.org>) and included:
ma-yvfgy for *Petromyzon marinus* CB1R (DOI:10.5452/ma-yvfgy),
ma-mi5fb for *Branchiostoma floridae* unclassified CBR (DOI: 10.5452/ma-mi5fb),
ma-dol4y for anandamide docked on cryo-EM *Homo sapiens* TRPV1 (DOI: 10.5452/ma-dol4y).
